# A Comparative Analysis of the Lipoprotein(a) and Low-Density Lipoprotein Proteomic Profiles Combining Mass Spectrometry and Mendelian Randomization

**DOI:** 10.1016/j.cjco.2020.11.019

**Published:** 2020-12-03

**Authors:** Raphaëlle Bourgeois, Arnaud Girard, Nicolas Perrot, Jakie Guertin, Patricia L. Mitchell, Christian Couture, Clarisse Gotti, Sylvie Bourassa, Paolo Poggio, Elvira Mass, Romain Capoulade, Corey A. Scipione, Audrey-Anne Després, Patrick Couture, Arnaud Droit, Philippe Pibarot, Michael B. Boffa, Sébastien Thériault, Marlys L. Koschinsky, Patrick Mathieu, Benoit J. Arsenault

**Affiliations:** a Centre de recherche de l’Institut universitaire de cardiologie et de pneumologie de Québec, Quebec, Canada; b Department of Medicine, Faculty of Medicine, Université Laval, Quebec, Canada; c Proteomics platform of the CHU de Québec, Quebec, Canada; d Centro Cardiologico Monzino IRCCS, Milan, Italy; e University of Bonn, Developmental Biology of the Immune System, Life and Medical Sciences Institute (LIMES), Bonn, Germany; f Université de Nantes, CHU Nantes, CNRS, INSERM, l’institut du thorax, Nantes, France; g Robarts Research Institute, Schulich School of Medicine and Dentistry, The University of Western Ontario, London, Ontario, Canada; h Centre de recherche du CHU de Québec, Quebec, Canada; i Department of Biochemistry, Schulich School of Medicine and Dentistry, The University of Western Ontario, London, Ontario, Canada; j Department of Molecular Biology, Medical Biochemistry and Pathology, Faculty of Medicine, Université Laval, Quebec, Canada; k Department of Surgery, Faculty of Medicine, Université Laval, Quebec, Canada

## Abstract

**Background:**

Lipoprotein(a) (Lp[a]), which consists of a low-density lipoprotein (LDL) bound to apolipoprotein(a), is one of the strongest genetic risk factors for atherosclerotic cardiovascular diseases. Few studies have performed hypothesis-free direct comparisons of the Lp(a) and the LDL proteomes. Our objectives were to compare the Lp(a) and the LDL proteomic profiles and to evaluate the effect of lifelong exposure to elevated Lp(a) or LDL cholesterol levels on the plasma proteomic profile.

**Methods:**

We performed a label-free analysis of the Lp(a) and LDL proteomic profiles of healthy volunteers in a discovery (n = 6) and a replication (n = 9) phase. We performed inverse variance weighted Mendelian randomization to document the effect of lifelong exposure to elevated Lp(a) or LDL cholesterol levels on the plasma proteomic profile of participants of the INTERVAL study.

**Results:**

We identified 15 proteins that were more abundant on Lp(a) compared with LDL (*serping1*, *pi16*, *itih1*, *itih2*, *itih3*, *pon1*, *podxl*, *cd44*, *cp*, *ptprg*, *vtn*, *pcsk9*, *igfals*, *vcam1*, and *ttr*). We found no proteins that were more abundant on LDL compared with Lp(a). After correction for multiple testing, lifelong exposure to elevated LDL cholesterol levels was associated with the variation of 18 plasma proteins whereas Lp(a) did not appear to influence the plasma proteome.

**Conclusions:**

Results of this study highlight marked differences in the proteome of Lp(a) and LDL as well as in the effect of lifelong exposure to elevated LDL cholesterol or Lp(a) on the plasma proteomic profile.

## Résumé

**Contexte:**

La lipoprotéine(a) (Lp[a]), qui est constituée d’une lipoprotéine de basse densité (LDL) liée à une apolipoprotéine(a), est l’un des plus importants facteurs de risque génétiques de survenue d’une maladie cardiovasculaire athéroscléreuse. Peu d’études comparatives directes sans hypothèse ont porté sur le protéome de la Lp(a) et celui des LDL. Nos objectifs étaient de comparer les profils protéomiques de la Lp(a) et des LDL et d’évaluer l’effet d’une exposition à vie à un taux élevé de Lp(a) ou de cholestérol LDL sur le profil protéomique plasmatique.

**Méthodologie:**

Nous avons réalisé une analyse sans marquage des profils protéomiques de la Lp(a) et des LDL chez des volontaires en bonne santé dans le cadre d’une phase de découverte (n = 6) et d’une phase de réplication (n = 9). Pour rendre compte de l’effet d’une exposition à vie à un taux élevé de Lp(a) ou de cholestérol des LDL sur le profil protéomique plasmatique des participants de l’étude INTERVAL, nous avons utilisé une analyse de randomisation Mendélienne avec pondération par l’inverse de la variance.

**Résultats:**

Nous avons relevé 15 protéines associées en plus grande abondance à la Lp(a) qu’aux LDL (*serping1*, *pi16*, *itih1*, *itih2*, *itih3*, *pon1*, *podxl*, *cd44*, *cp*, *ptprg*, *vtn*, *pcsk9*, *igfals*, *vcam1* et *ttr*). Nous n’avons noté aucune protéine associée en plus grande abondance aux LDL qu’à la Lp(a). Après correction pour tenir compte de la multiplicité des tests, l’exposition à vie à un taux élevé de cholestérol LDL a été associée à la variation de 18 protéines plasmatiques, tandis que le taux de Lp(a) ne semblait pas influencer le protéome plasmatique.

**Conclusions:**

Les résultats de notre étude font ressortir les différences marquées entre le protéome de la Lp(a) et celui des LDL, ainsi qu’entre l’effet sur le profil protéomique plasmatique de l’exposition à vie à un taux élevé de cholestérol LDL et celui de l’exposition à vie à un taux élevé de Lp(a).

Circulating levels of apolipoprotein (apo) B-containing lipoproteins such as low-density lipoprotein (LDL) and lipoprotein(a) (Lp[a]) are causal risk factors for a broad range of atherosclerotic cardiovascular diseases (ACVD).^1^ The evidence linking LDL cholesterol levels with ACVD risk emerged from experimental preclinical studies, follow-up of patients with inherited lipid disorders associated with lifelong exposure to elevated concentrations of LDL cholesterol levels, and most importantly, cardiovascular outcomes trials of LDL cholesterol-lowering documenting important cardiovascular benefits of pharmacological interventions aimed at reducing LDL cholesterol levels. Although LDL cholesterol levels are dose-dependently associated with cardiovascular events in the general population, many cardiovascular events occur in people with untreated LDL cholesterol levels in the normal range whereas some individuals with high LDL cholesterol levels are free of ACVD. Although this observation could be attributable to the presence/absence of other established coronary artery disease risk factors, such a discrepancy could also be attributable to interindividual variability in LDL particle physical and chemical properties. LDL particles are heterogeneous in terms of size, density, number, lipid cargo, and protein composition. Smaller and denser LDL particles predict the risk of cardiovascular disease in the general population.^2^ In addition to physical characteristics such as size and density, the composition of LDL can be very heterogeneous across the population and the LDL proteome and/or lipidome might be altered in patients with cardiometabolic disorders.^3^

Lp(a) is characterized by an apo(a) molecule associated with an LDL particle by a disulphide bridge linking LDL apoB to apo(a).^4^ The proatherogenic effects of Lp(a) could be due to the LDL and apo(a) moiety of Lp(a), because the latter has been shown to promote thrombosis and inflammation.^5^ Apo(a) has also been shown to inhibit hepatitis C virus invasion through interaction with infectious particles.^6^ Proteins other than apo(a) or apoB might also be carried by Lp(a) in the plasma, some of which were previously identified by von Zychlinski et al.^7^ It appears that the role of these newly identified proteins extends beyond lipoprotein metabolism and might include wound healing and complement activation. We also reported that Lp(a)-bound autotaxin (*atx*) promotes mineralization and inflammation of the aortic valve^8^^,^^9^ and this increased plasma *atx* activity associated with Lp(a) levels is predictive of calcific aortic valve stenosis (CAVS) risk.^10^ In addition to being some of the first to report on the molecular mechanisms through which Lp(a) causes aortic valve calcification or CAVS, these studies suggest that in addition to promoting CAVS and inflammation through its oxidized phospholipids (OxPL) content, the proatherogenic properties of Lp(a) could be mediated by proteins and enzymes carried by this unique lipoprotein. It was also recently reported that *atx* transported by Lp(a) was significantly associated with ATX transported by apoB in the human plasma and that *atx* could be detected in advanced aortic valve lesions rich in cholesterol crystals.^11^ Although a targeted proteomic analysis of 17 proteins known to be transported by either LDL or Lp(a) revealed potential differences in the signature of the proteome of LDL vs Lp(a),^12^ few studies, if any, performed direct comparisons of the Lp(a) and the LDL proteomes and in no study, to our knowledge, have the LDL and Lp(a) proteomic profiles been compared in the same individuals.

Plasma proteins are important determinants of health and disease. Although RNA interference therapies aimed at lowering Lp(a) levels are currently under investigation for their potential benefits on cardiovascular outcomes, the effect of these therapies on the plasma proteome is currently unknown. Additionally, whether exposure to elevated levels of LDL cholesterol levels influences the plasma proteome is currently unknown. Mendelian randomization (MR) enables the assessment of the effect of lifelong exposure to biological traits on outcomes.^13^ This technique takes advantage of the use of genetic variants (which are less susceptible to confounding compared with directly measured exposures) associated with traits of interest (such as Lp[a] and LDL cholesterol levels) as instrumental variables to determine whether these exposures might be causally linked with an outcome (such as the plasma proteomic profile).

The objectives of the present study were twofold. First, we aimed at performing a comprehensive characterization and comparison of the proteome of Lp(a) and LDL particles isolated in healthy individuals. Second, we evaluated the effect of lifelong exposure to elevated Lp(a) or LDL cholesterol levels on the human plasma proteomic profile using MR.

## Methods

### Study participants

We isolated Lp(a) and LDL fractions from 6 healthy individuals (3 men and 3 women; discovery phase) and from 9 healthy individuals (5 men and 4 women; replication phase). All individuals had Lp(a) levels ≥ 125 nmol/L. Isolated LDL particles were also obtained from healthy individuals (3 men and 3 women) with very low Lp(a) levels (10.5 ± 5.1 nmol/L) using the same method. Plasma Lp(a) levels were measured using a turbidimetric assay using the Tina-quant Lipoprotein(a) Gen.2 system (Cobas Integra 400/800, Roche Diagnostics, Mannheim, Germany). Each participant included in this study signed a consent form approved by the Institut universitaire de cardiologie et de pneumologie de Québec ethics review board.

### Assessment of the Lp(a) and LDL proteomes using nanoLC-MS/MS

Lp(a) and LDL were isolated using a combination of ultracentrifugation and fast protein liquid chromatography, as previously described.^9^^,^^14^ Briefly, fresh serum was ultra centrifuged at 4°C with an iodixanol (Optiprep; BioVision Inc, Milpitas, CA) solution, leading to differentially enriched fractions. The Lp(a) enriched fraction, assessed using Sebia Hydragel lipo + Lp(a) (Sebia, Lisses, France) was then submitted to size exclusion chromatography and apo(a)-positive fractions (controlled with Western blot analysis) were submitted to affinity chromatography with an NaCl gradient to further purify Lp(a). After affinity chromatography, Lp(a) and LDL samples were dialyzed and filtered, and final purity assessed with Sebia Hydragel Lp(a). Isolated fractions were then precipitated overnight with acetone at −20°C. When resuspended, samples were reduced and alkylated then digested to completion with trypsin. Resulting peptides were analyzed using nanoscale liquid chromatography combined to tandem mass spectrometry (nanoLC-MS/MS) with a Dionex UltiMate 3000 RSLCnano chromatography system (Thermo Fisher Scientific, Waltham, MA) connected to an Orbitrap Fusion Tribrid mass spectrometer (Thermo Fisher Scientific). Peptides (2 μg) were trapped at 20 μL/min in loading buffer (2% acetonitrile, 0.05% trifluoroacetic acid) on a precolumn for 5 minutes followed by separation on a Pepmap Acclaim 50 cm × 75 column (ThermoFisher Scientific) equilibrated in 95% solvent A (2% acetonitrile (ACN)/0.1% formic acid) and 5% solvent B (80% ACN/0.1% FA). Peptides were then eluted with a 5% to 40% of solvent B gradient for 90 minutes (120 minutes run), at 300 nL/min. Mass spectra were acquired using a data-dependent acquisition mode using Thermo Xcalibur software version 3.0.63 (ThermoFisher Scientific). Full scan mass spectra (350-1800 m/z) were acquired in the orbitrap at a resolution of 120,000. Every intense precursor ion detected for 3 seconds (top speed mode) was selected to be fragmented using higher-energy collisional dissociation and fragment ions were detected in the linear trap. A dynamic exclusion of precursor ions of 20 seconds was applied to avoid redundant selection of peptides.

Spectra were searched against the Uniprot complete proteome homo sapiens database (https://www.uniprot.org/help/homo_sapiens) using the Andromeda module of MaxQuant software version 1.6.0.16 (Max-Planck-Institute of Biochemistry, Martinsried, Germany). Trypsin/P enzyme parameter was selected with 2 possible missed cleavages. Carbamidomethylation of cysteine was set as fixed modification whereas methionine oxidation was set as variable modifications. Mass search tolerance were 5 ppm and 0.5 Da for mass spectrometry and tandem mass spectrometry, respectively. For protein validation, a maximum false discovery rate of 1% at peptide and protein level was used on the basis of a target/decoy search. The raw intensity values extracted using MaxQuant (Max-Planck-Institute of Biochemistry) in each sample replicate were normalized using the median value and were used to calculate the ratio between the 2 conditions. When intensity values were missing, they were replaced by a noise value corresponding to the first percentile of intensity values of all proteins of the sample replicate. A protein was considered to be quantifiable only if at least 67% replicate values (4 of 6 samples) were present in 1 of the compared samples [LDL or Lp(a)] with at least 2 peptides per protein. Statistical analyses were conducted on a mean Lp(a) intensity/mean LDL intensity ratio with paired sample *t* test with corrected *P* value (Benjamini-Hochberg) to adjust for the false discovery rate. Linear models for microarray data (Limma in R [The R Project for Statistical Computing; Vienna, Austria]) were carried out to adjust for multiple testing. Data analyses were conducted with RStudio 1.1.383 (RStudio, PBC, Boston, MA).

### Assessment of *apob* and *pcsk9* in lipoproteins using parallel reaction monitoring

Absolute quantification of *apob* and *pcsk9* in Lp(a) and LDL fractions was assessed by targeted parallel reaction monitoring (PRM) experiments on the Orbitrap Fusion Tribrid mass spectrometer (ThermoFisher Scientific). Isolated fractions of Lp(a) and LDL were prepared as described for the nanoLC-MS/MS experiment and samples were then resuspended in 2% ACN/0.05% trifluoroacetic acid to a final concentration of 0.5 μg/μL.

C-ter lysine labelled peptides ATLYALSHAVNNYH[^13^C_6_-^15^N_2_]K and ILHVFHGLLPGFLV[^13^C_6_-^15^N_2_]K (AQUA peptides, Pierce; Thermo Fisher Scientific) were respectively synthetized for ApoB and *PCSK9*. Standard curves were prepared by the addition of 5 different and controlled amounts of heavy peptides into 2 μg of pooled Lp(a) of LDL samples (from 10 to 1000 fmol/μL and from 50 to 5000 fmol/μL for *apob* peptide, respectively in Lp(a) and LDL samples, and from 0.4 to 40 fmol/μL for *PCSK9* peptides for Lp(a) and LDL samples). Before injection, 8 fmol of commercial CytoC digest (Thermo Fisher Scientific) was also added as internal standard and each standard was injected twice. Then, 2 μg of Lp(a) or LDL samples were spiked with 8 fmol of CytoC digest (Thermo Fisher Scientific), 20 fmol of *pcsk9* heavy peptide, and 600 fmol and 3000 fmol of *apob* heavy peptide, respectively, for Lp(a) and LDL samples. Peptide signal extraction was done with Skyline software,^15^ and the area from the 6 most abundant transitions per peptide were used for quantification. The average for the 2 injection replicates was then performed, and endogenous signal was normalized with the corresponding internal heavy standard. For each peptide, a mean ratio (Lp[a]/LDL) and a Welch *P* value were calculated.

### MR analyses

We used data from the human protein atlas, which includes genome-wide association study summary statistics of 2994 plasma proteins measured in 3301 participants from the INTERVAL study using an aptamer-based multiplex protein assay (SOMAscan; SomaLogic, Boulder, CO).^16^ Genome-wide association study summary statistics of 41 circulating cytokines and growth factors, obtained from the study of Ahola-Olli et al.^17^ were also used. In this study, plasma proteins were measured using premixed Bio-Plex Pro Human Cytokine 27-plex Assay and 21-plex Assay (Bio-Rad, Hercules, CA) in 8293 participants from the Cardiovascular Risk in Young Finns Study and Finland Cardiovascular Risk Study surveys. We used the inverse variance-weighted (IVW) MR (IVW-MR) to assess the effect of lifelong exposure to elevated Lp(a) and elevated LDL cholesterol levels on the plasma proteomic profile as implemented in the R package MendelianRandomization (https://rdrr.io/cran/MendelianRandomization). To obtain genetic estimates, IVW-MR performs a meta-analysis of each Wald ratio (the ratio of the effect of the genetic instrument on Lp[a]/LDL cholesterol levels and its effect on the plasma proteome). Our genetic instrument for Lp(a) includes 26 independent single nucleotide polymorphisms (SNPs), weighted for the effect on Lp(a) (per 1 mg/dL) as previously described.^18^^,^^19^ The LDL cholesterol genetic instrument was obtained from the Global Lipids Genetic Consortium.^20^ We used variants that passed the level of genome-wide significance for LDL cholesterol levels (< 5 × 10^−8^) and performed linkage disequilibrium-clumping to identify independent SNPs (*R*^2^ < 0.1). Our genetic instrument for LDL cholesterol includes 54 independent SNPs, weighted for the effect on LDL cholesterol (per 1 SD increment). We considered proteins to be statistically influenced by exposure to high Lp(a) or LDL cholesterol after correction for multiple testing (*P* = 0.05/3283 aptamer-targeted proteins = 1.5 × 10^−5^ for the INTERVAL cohort and *P* = 0.05/41 proteins = 1.2 × 10^−3^ for the Cardiovascular Risk in Young Finns Study and FINRISK survey).

## Results

### Label-free analysis of the Lp(a) and LDL proteomic profiles

**Figure 1:**
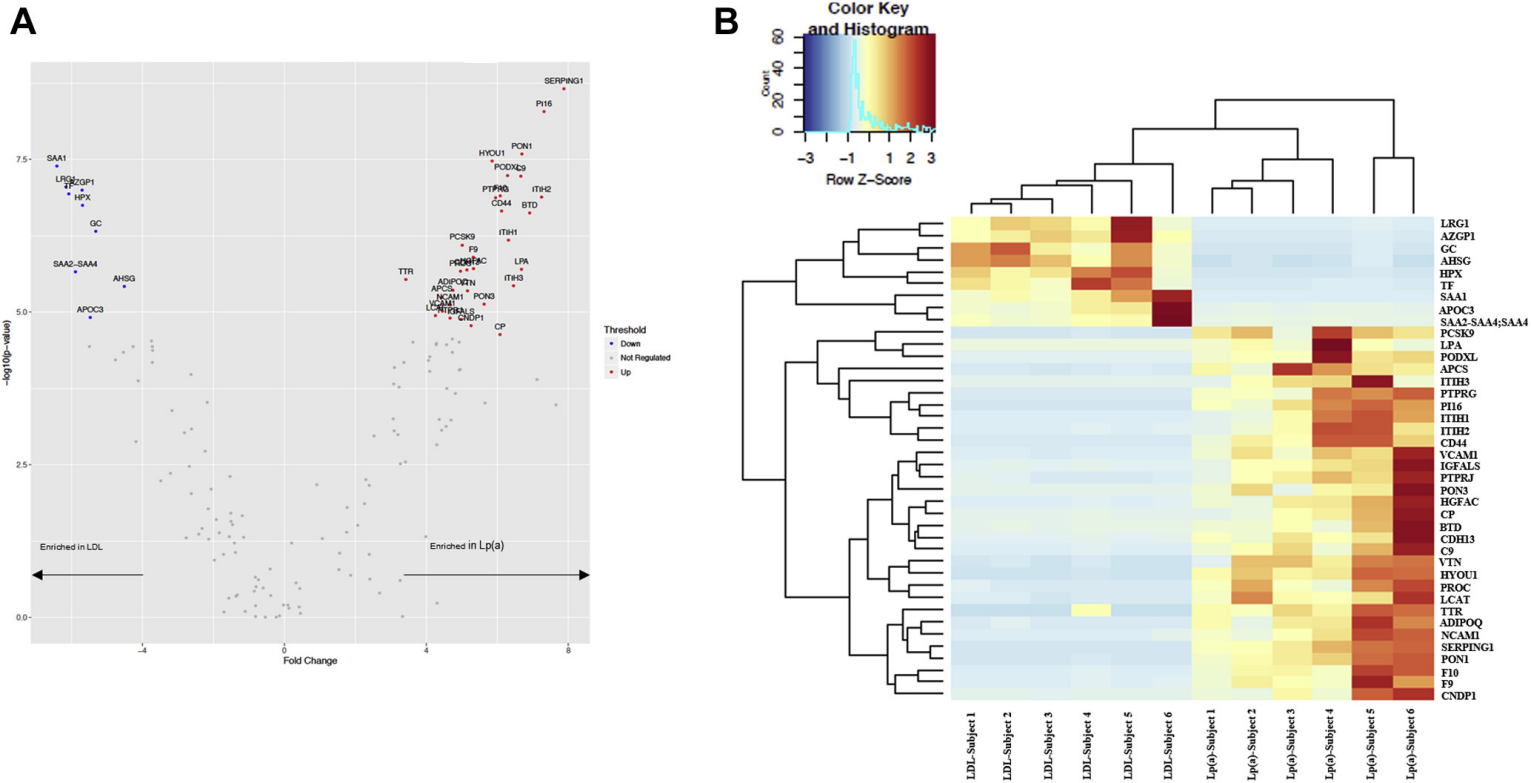
Discovery phase proteomic analysis. Volcano plot (**A**) and (**B**) heat map establishing correlations between low-density lipoprotein and lipoprotein(a) samples and the abundance of the variable proteins. Cold and hot colours represent low and high correlation levels, respectively. Changes in protein levels were determined by limma-corrected q value < 0.0001. Data are presented with hierarchical clustering in rows and columns.

**Figure 2:**
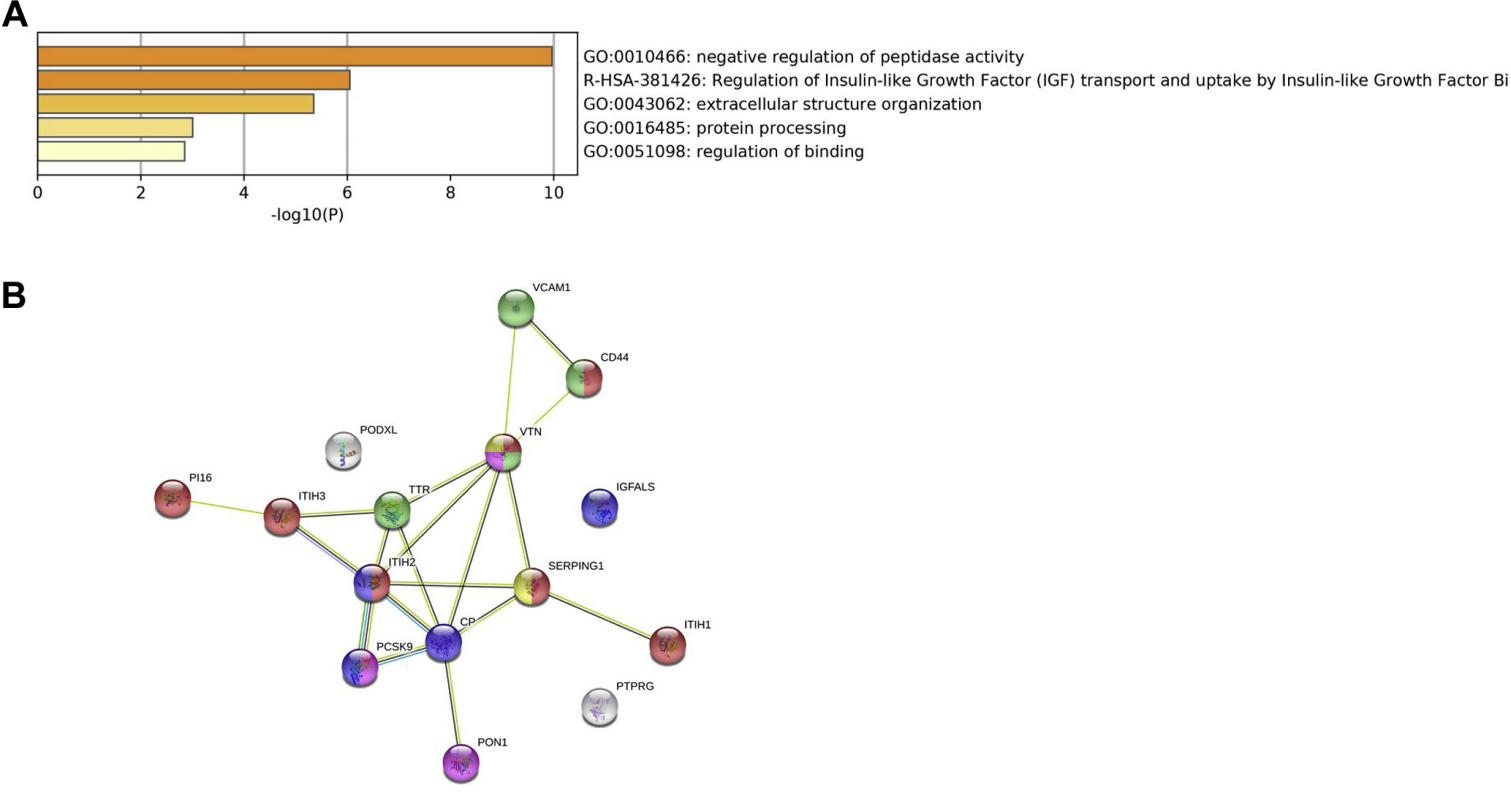
(**A**) Functional enrichment analysis and (**B**) metabolic pathway analysis using String db of the proteins identified as preferentially associated with lipoprotein(a) in discovery and replication phases. **Red** represents proteins involved in the negative regulation of peptidase activity, **blue** the proteins involved in regulation of IGF transport and uptake by IGFB, **green** the proteins involved extracellular matrix organization, **purple** the proteins involved in the regulation of binding, and **yellow** the proteins involved in protein processing. IGF, insulin-like growth factor; IGFB, insulin-like growth factor-binding protein.

In the discovery phase, 154 proteins were identified in the LDL and/or Lp(a) fractions. A total of 40 proteins were statistically differentially enriched: 9 proteins were preferentially associated with LDL compared with Lp(a) and 31 were preferentially enriched in Lp(a) compared with LDL (Fig. 1A). Heat map representation of the results (Fig. 1B) shows a heterogenic repartition of the proteins identified and quantified between the 2 conditions LDL and Lp(a). A total of 15 proteins were replicated in the replication phase for every sample. These 15 proteins were preferentially associated with Lp(a) particles compared with LDL. None of the proteins preferentially associated with LDL compared with Lp(a) in the discovery phase were replicated (Table 1). Enrichment pathway analysis performed with Metascape (https://metascape.org; Fig. 2A) showed that these 15 proteins were involved in negative regulation of peptidase activity (GO:0010466, *P* = 1.07 × 10^−10^), regulation of insulin growth factor, and transport by insulin growth factor (R-HSA-381426, *P* = 8.91 × 10^−7^), extracellular structure organization (GO:0043062, *P* = 4.47 × 10^−6^) protein processing (GO:0016485, *P* = 0.001) and regulation of binding (GO:0051098, *P* = 0.001). By using the 15 proteins that were replicated, we extracted a network from STRING v11 (https://string-db.org), a curated database of direct and indirect (functional) protein-protein interactions. The network included 15 nodes and 19 edges (Fig. 2B) showing multiple interactions between 12 of the 15 proteins. This analysis suggests that these proteins might jointly contribute to a shared function.

**Table 1:** Identification of proteins associated with lipoprotein(a) and low-density lipoproteins fractions.

Gene	Protein	Discovery phase		Replication phase	
		Lp(a)/LDL ratio	*P*	Lp(a)/LDL ratio	*P*
*SERPING1*	Plasma protease C1 inhibitor	234.8	2.19 × 10^−9^	108.1	1.65 × 10^−5^
*PI16*	Peptidase inhibitor 16	158.8	5.19 × 10^−9^	17.2	0.02
*ITIH2*	Inter-alpha-trypsin inhibitor heavy chain H2	151.5	1.30 × 10^−7^	17.8	1.14 × 10^−5^
*PON1*	Serum paraoxonase/arylesterase 1	103.4	2.59 × 10^−8^	35.0	2.39 × 10^−6^
*ITIH3*	Inter-alpha-trypsin inhibitor heavy chain H3	87.8	3.69 × 10^−6^	30.2	9.39 × 10^−4^
*ITIH1*	Inter-alpha-trypsin inhibitor heavy chain H1	79.3	6.57 × 10^−7^	16.0	2.74 × 10^−5^
*PODXL*	Podocalyxin	77.9	5.74 × 10^−8^	150.9	6.53 × 10^−10^
*CD44*	CD44 antigen	69.3	2.22 × 10^−7^	253.0	5.80 × 10^−11^
*CP*	Ceruloplasmin	67.5	2.31 × 10^−5^	614.7	1.16 × 10^−5^
*PTPRG*	Receptor-type tyrosine-protein phosphatase gamma	61.8	1.32 × 10^−7^	62.1	1.40 × 10^−8^
*VTN*	Vitronectin	35.7	4.50 × 10^−6^	9.8	6.71 × 10^−6^
*PCSK9*	Proprotein convertase subtilisin/kexin type 9	32.3	7.94 × 10^−7^	6.7	1.66 × 10^−2^
*IGFALS*	Insulin-like growth factor-binding protein complex acid labile subunit	31.4	1.31 × 10^−5^	6.5	2.93 × 10^−5^
*VCAM1*	Vascular cell adhesion protein 1	21.9	9.62 × 10^−6^	5.0	1.61 × 10^−2^
*TTR*	Transthyretin	10.7	2.89 × 10^−6^	16.2	1.89 × 10^−7^

LDL, low-density lipoprotein; Lp(a), lipoprotein(a).

### Comparison with isolated LDL particles of individuals with low Lp(a) levels

**Figure 3:**
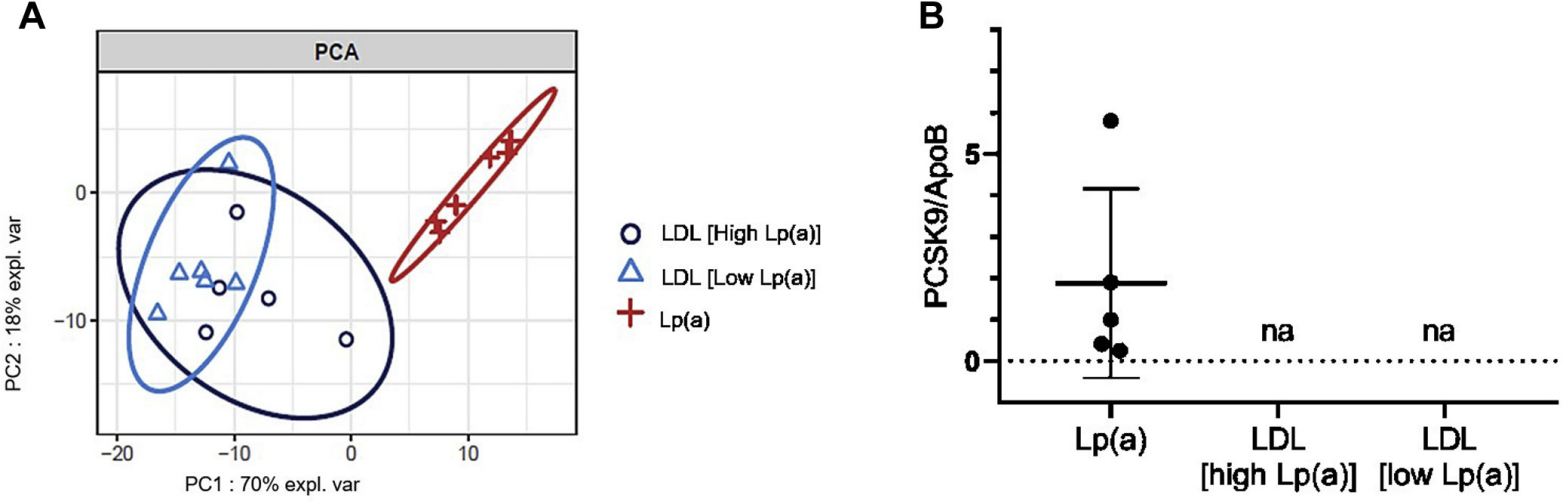
PCA analysis of the (**A**) proteomic composition and (**B**) parallel reaction monitoring (PRM) analysis of *pcsk9* and apolipoprotein B (*apob*) in Lp(a) and LDL of subjects with high vs low Lp(a). *Pcsk9* and *apob* concentrations are expressed in fmol/ug of the total sample. LDL, low-density lipoprotein; LP(a), lipoprotein(a); PCSK9, proprotein Convertase Subtilisin/Kexin type 9.

The Lp(a) proteome seems richer than the LDL proteome, in the context of high Lp(a) levels. To confirm that the Lp(a) proteome is specific and not redistributed among LDL particles in a context of low Lp(a), we performed additional semiquantitative PRM analysis of the 15 proteins identified previously in LDL fractions from 6 subjects with very low Lp(a) levels. Principal component analysis (Fig. 3A) showed that the proteome of LDL is similar in the context of either high or low Lp(a), because the Lp(a) proteome is clearly distinguishable from the others. Results of this analysis suggests that the proteins associated with Lp(a) might not be redistributed in a context of low Lp(a) and that this proteomic signature is specific of Lp(a). At the same time, because LDL and Lp(a) particles each contain one apoB molecule per particle, we performed quantitative PRM of apoB and *pcsk9* in these samples. Results of this analysis showed that *pcsk9* was only detectable on Lp(a) fractions, even when compared with LDL particles of the same subjects and from LDL particles from the individuals with low Lp(a) levels, suggesting higher abundance of *pcsk9* in Lp(a) compared with LDL. Figure 3B shows the ratio between the concentration of *pcsk9* and the concentration of apoB, both concentrations being in fmol/μg of either the LDL or Lp(a) sample.

### MR analyses

**Figure 4:**
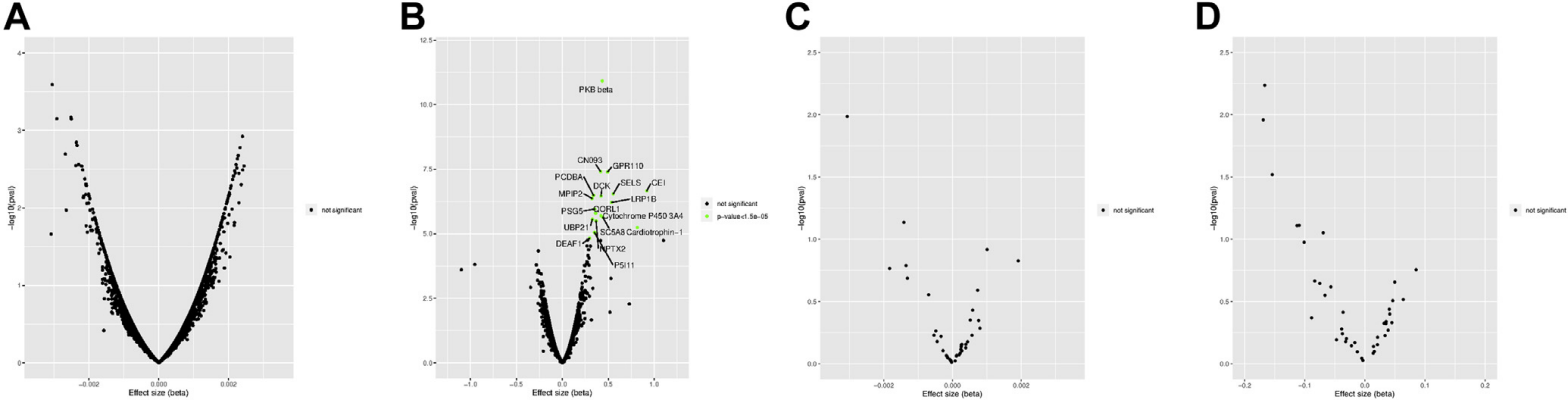
Volcano plot of plasma proteins significantly influenced by lifelong exposure to (**A**) elevated Lp(a) or (**B**) LDL cholesterol levels and cytokines and growth factors influenced by lifelong exposure to (**C**) elevated Lp(a) or (**D**) LDL cholesterol levels. LDL, low-density lipoprotein; Lp(a), lipoprotein(a).

Figure 4 shows volcano plots of all proteins that were significantly influenced by lifelong exposure to elevated Lp(a) (Fig. 4A) or LDL cholesterol levels (Fig. 4B). This analysis shows that after correction for multiple testing, no proteins appeared to be influenced by Lp(a) whereas 18 proteins might be influenced by elevated LDL cholesterol levels. These 18 proteins are described in Table 2. This table also presents the 18 proteins that might be more strongly influenced by Lp(a), even if these did not pass multiple testing correction. Because several proteins involved in proinflammatory pathways were identified in the Lp(a) fraction (Fig. 2), we also investigated the association between exposure to elevated Lp(a) (Fig. 4C) or LDL cholesterol levels (Fig. 4D) and a panel of 41 inflammatory cytokines and growth factors. However, after correction for multiple testing, no associations were found.

**Table 2:** Mendelian randomization analysis of circulating proteins associated with lifelong exposure to elevated Lp(a) and LDL levels

Protein	β	SE	*P*
Lp(a)			
Ig K chain V-I region HK102-like	−0.0030	0.0008	0.0003
ICOS	−0.0025	0.0007	0.0007
LRP1B	−0.0029	0.0009	0.0007
Fas. soluble	−0.0025	0.0007	0.0007
HGD	0.0024	0.0007	0.0012
Fas, soluble	−0.0024	0.0007	0.0014
Trefoil factor 2	−0.0023	0.0007	0.0016
NMT1	0.0023	0.0007	0.0017
SC5A8	−0.0027	0.0009	0.0020
Fragile histidine triad protein	0.0023	0.0007	0.0021
SAPL1	0.0023	0.0007	0.0023
CDON	−0.0023	0.0008	0.0027
SYAC	0.0022	0.0007	0.0028
BAG3	−0.0024	0.0008	0.0028
ERAB	0.0024	0.0008	0.0029
TNF SR-II	−0.0022	0.0007	0.0029
Glutathione S-transferase Pi	0.0022	0.0008	0.0030
IL-12 RB2	−0.0022	0.0007	0.0032
LDL			
PKB B	0.4349	0.0642	1.22 × 10^−11^
CN093	0.4163	0.0757	3.88 × 10^−8^
GPR110	0.4928	0.0898	4.04 × 10^−8^
CEI	0.9195	0.1774	2.18 × 10^−7^
SELS	0.5541	0.1080	2.89 × 10^−7^
PCDBA	0.3396	0.0665	3.25 × 10^−7^
DCK	0.4210	0.0826	3.47 × 10^−7^
MPIP2	0.3240	0.0641	4.30 × 10^−7^
LRP1B	0.5355	0.1074	6.11 × 10^−7^
PSG5	0.3503	0.0719	1.12 × 10^−6^
QORL1	0.3625	0.0755	1.60 × 10^−6^
Cytochrome P450 3A4	0.4206	0.0885	2.01 × 10^−6^
SC5A8	0.4386	0.0928	2.26 × 10^−6^
UBP21	0.3271	0.0698	2.76 × 10^−6^
NPTX2	0.3677	0.0790	3.20 × 10^−6^
Cardiotrophin-1	0.8167	0.1801	5.76 × 10^−6^
P5I11	0.3508	0.0790	9.07 × 10^−6^
DEAF1	0.2974	0.0687	1.49 × 10^−5^

All 18 proteins significantly influenced by LDL cholesterol levels after correction for multiple testing are shown as well as the first nominally significant proteins influenced by Lp(a).

Ig, immunoglobulin; LDL, low-density lipoprotein; Lp(a), lipoprotein(a).

## Discussion

In light of the growing body of evidence supporting a causal role for Lp(a) particles in the pathobiology of ACVD and of the development of Lp(a)-lowering therapies to lower the risk of ACVD, we set out to gain more knowledge on the physico-chemical characteristics of Lp(a) such as its proteome using semiquantitative proteomics and the potential effect of Lp(a)-lowering therapy on the plasma proteome using MR. Results of the present study suggest that the proteome of Lp(a) is substantially richer and more diverse than that of LDL. Our unbiased approach in comparing the proteome of Lp(a) with that of LDL isolated from the same individuals in a discovery and a replication phase identified 15 proteins that were preferentially associated with Lp(a) compared with LDL. Investigating whether lifelong exposure to elevated Lp(a) or LDL cholesterol levels influenced the plasma proteome, we found that exposure to higher LDL cholesterol levels likely has a more important effect on the plasma proteome than exposure to high Lp(a) levels. These results suggest that although Lp(a) particles might carry several proteins, exposure to high Lp(a) likely has a trivial effect globally on the plasma proteome.

In a previous study von Zychlinski et al.^7^ identified proteins potentially transported by Lp(a). Interestingly, the role of these proteins extends beyond lipoprotein metabolism and could be involved in wound healing and complement activation. Their results also suggest that Lp(a) might carry more apoA1, apoA2, apoC1, apoC3, apoD, apoF, and clusterin while carrying less apoM than LDL particles. Another study also suggested that Lp(a) could carry the proinflammatory cytokine monocyte chemoattractant protein-1 (*mcp1*).^21^ Another targeted proteomic analysis identified 17 proteins transported by Lp(a).^12^ Our results provide confirmation that transthyretin, vitronectin (*vtn*), and paraoxonase-1 and protease inhibitors might be preferentially transported by Lp(a) compared with LDL. One important caveat of these studies is that Lp(a) and LDL particles that were compared were not obtained from the same study participants, which is in contrast to our investigation, in which we compared LDL and Lp(a) isolated from the same participants (all with Lp[a] ≥ 125 nmol/L). This strategy enabled us to perform matched analyses, but more importantly, results emerging from this approach provides important differences in the Lp(a) and LDL proteome that are not subject to interindividual differences in the proteome of these lipoproteins, and might explain why some of the proteins identified in these studies were not found in our study. Our analytical framework identified 15 proteins that are more abundant on Lp(a) compared with LDL and no proteins were found to be more present on LDL compared with Lp(a) after the replication phase. Although we used the exact same procedure for the isolation, separation, and purification of Lp(a) and LDL particles, and performed experiments in parallel for Lp(a) and LDL isolated from the same study participants, a systematic methodological bias in the treatment of these subfractions cannot be entirely ruled out. However, because Lp(a) and LDL particles only differ by the presence of apo(a) on Lp(a), protein binding to the apo(a) moiety of Lp(a) could explain, at least in part, why the proteome of Lp(a) is richer than that of LDL. This hypothesis is supported by recent studies showing specific binding of ATX to the apo(a) moiety of Lp(a)^9^ and lack of binding of *mcp1* to Lp(a) after incubation of Lp(a) with the E06 antibody (which targets the OxPL-binding site of apo[a]).^21^ Apo(a) also directly binds to foam cell receptors.^22^

Proteins transported by Lp(a) might be involved in several biological pathways such as the acute inflammatory response, extracellular structure organization, and protease inhibition, which might explain to some extent why Lp(a) particles contribute to inflammation in the vascular endothelium.^23^ This study also suggests that Lp(a) could have proinflammatory properties. Indeed, exposure of monocytes to Lp(a) increased cytokine production and accelerated monocyte influx. Similar observations were also reported *ex vivo* in patients with Lp(a) levels > 50 mg/dL. Interestingly, in the same report these patients were also shown to have higher arterial wall inflammation assessed using single-photon emission computed tomography.^23^ Whether the inflammatory response could be triggered by components of the Lp(a) proteome, in addition to its OxPL content, needs to be studied further.

In support of the “inflammatory proteome” of Lp(a), studies have shown that, ceruloplasmin (*cp*), a specific copper plasma glycoprotein, involved in membrane stability and immune response, leads to an increase in LDL oxidation^24^ and its nondegraded form has been identified as a risk factor for coronary artery disease.^25^ Higher *cp* levels were also observed in subjects with myocardial infarction, atherosclerosis, or aneurysm.^26^ However, *cp*-bound copper seems to influence the oxidative activity of the protein, as pro-oxidant activity is lost when a single copper is depleted.^24^ Additionally, selectins, integrins, and immunoglobulins have an important role in the macrophage recruitment process, involving vascular cell-adhesion molecule-1 (*vcam1*). *vcam1* mediates adhesion of leucocytes^27^ such as lymphocytes, monocytes, and eosinophils.^28^^,^^29^ Interestingly, it was reported that *vcam1* is increased in the early stages of atherosclerosis,^30^^,^^31^ and elevated levels of *vcam1* are correlated with subclinical atherosclerotic disease.^32^ In addition to *vcam1*, another Lp(a)-associated protein that is involved in cell adhesion is *vtn*. It was shown that subjects with 2 or more stenotic arteries have higher *vtn* levels.^33^ *Vtn* also promotes neointima development by enhancing vascular smooth muscle cell migration, thus promoting atherosclerosis. Members of the inter-alpha-trypsin inhibitors (ITIH) family (*itih1*, *itih2*, and *itih3* in this study) are also involved in cell adhesion and migration; they allow the formation of a cable structure with hyaluronic acid in inflammation sites, by enhancing leucocyte migration.^34^^,^^35^ Podocalyxin is also involved in adhesion and cellular migration^36^^,^^37^ and could possibly play a role in atherosclerosis. *Cd44* has a critical role in the recruitment of leukocytes and is upregulated and functionally activated during inflammation,^38^ and is potentially important for the recruitment of macrophages and their retention in atherosclerosis.^39^^,^^40^ *Cd44* is also linked to calcium deposition after osteopontin binding, which is of potential pathophysiological relevance because of the important role of Lp(a) in CAVS.^41^

Metalloproteinases facilitate endothelial smooth cell migration from media to intima, then leading to wall vessel thickening. Interestingly, Peptidase inhibitor 16, a serine peptidase inhibitor, has been reported to decrease the expression of metalloproteinases,^42^ and to be increased in cardiovascular disease.^43^ It is also upregulated in coronary artery endothelial cells under laminar flow with elevated shear stress. In our analysis Serpin G1 was the most abundant protein on Lp(a). This protease inhibitor is also involved in several pathways in the cascade system, such as the complement system as well as coagulation and fibrinolytic systems.^44^

Hypothesis-driven studies have shown that *pcsk9* could be transported by LDL and Lp(a),^45^^,^^46^ but no one, to our knowledge, has sought to determine whether Lp(a) or LDL was the preferential carrier of *PCSK9* in these lipoprotein fractions isolated from the same individuals. Our study, to our knowledge, is the first hypothesis-free study to identify *pcsk9* on Lp(a). Further, our results confirm the work of Tavori et al.,^46^ who have shown that *pcsk9* was present on Lp(a) but not on LDL using a different method. Although it has been shown that *pcsk9* inhibition could reduce Lp(a) levels by up to 30%,^47^ it remains to be determined if treatment with antibodies targeted at *pcsk9* reduced Lp(a) levels by targeting *pcsk9*-Lp(a) complexes.

Although our proteomic analyses of isolated lipoprotein fractions suggest that the proteome of Lp(a) particles is somewhat richer than that of LDL particles, our MR analyses showed that lifelong exposure to elevated LDL cholesterol concentrations had a more important effect on the plasma proteome than high or low Lp(a) levels. Interestingly, we found that LDL receptor related protein 1B (*lrp1b*), a protein that has been suggested to be transported by apoE-containing lipoproteins^48^ was higher in participants characterized by exposure to higher LDL cholesterol levels whereas *lrp1b* concentrations were lower in patients characterized by exposure to higher Lp(a) particles. These findings are of potentially interest because no detrimental effect on the plasma proteome should be expected from Lp(a)-lowering therapies.

Our study has limitations. For instance, although the semiquantitative approach that was used enable us to interrogate the Lp(a)/LDL proteome in an unbiased manner, we could not determine the proportion of each protein that is carried by Lp(a). Additionally, although we used a discovery and a validation phase in 2 separate study samples, the number of participants included in this study is relatively low and further studies will be required to comprehensively characterize the proteome of these lipoprotein subfractions. Finally, our MR analyses identified circulating proteins that might be influenced by exposure to either high Lp(a) or high LDL levels. It is unsure however, if these proteins track with Lp(a)/LDL because they are carried by these lipoproteins or because of secondary mechanisms.

In conclusion, we performed a detailed characterization of the Lp(a) proteome and compared it with that of LDL. In our study the proteome of Lp(a) was richer and more diversified than the LDL proteome. Upon characterization of the Lp(a) proteome, we also noticed that the Lp(a) proteome included several proteins associated with poor cardiovascular outcomes. Our results, which will need to be replicated in healthy individuals and in patients with and without ACVD, provide valuable information on the biological pathways that link Lp(a) to ACVD risk. Despite the fact that Lp(a) transports several proteins in the bloodstream, as in our study, the effect of lifelong exposure to high Lp(a) on the plasma proteome appears to be small. However, these molecules could amplify the deleterious effects of Lp(a) by enhancing their delivery to sites of vessel wall injury or aortic valve tissue. Whether or not Lp(a) inhibitors will have a role in reducing residual ACVD risk and whether the reduction in Lp(a)-associated molecules will play a role will ultimately need to be tested in large-scale intervention studies.

## Funding Sources

This study was supported by grants from the Canadian Institutes of Health Research awarded to B.J.A. (FRN149068 and FRN155226) and by the Fondation de l’IUCPQ. This study was also supported by the European Research Area Network on Cardiovascular Disease Joint Transnational Call 2018 (PICASSO JTC2018-042), which is a European Research Area Network comprising 24 partners from 19 countries/regions that has been granted for funding through the current EU Framework Programme for Research and Innovation “Horizon 2020.” A.-A.D. is supported by a master’s training award from the Fonds de recherche du Québec: Santé (FRQS). N.P. is supported by a doctoral training award from the FRQS. P.M. holds a FRQS Research Chair on the Pathobiology of Calcific Aortic Valve Disease. M.L.K. is supported by a grant from the Heart and Stroke Foundation of Canada (G-17-0018740) for this work. P.P. holds the Canada Research Chair in Valvular Heart Disease and his research program is supported by a Foundation Scheme Grant from the Canadian Institutes of Health Research. R.C. is supported by a “Connect Talent” research chair from Région Pays de la Loire and Nantes Métropole. B.J.A. and S.T. hold junior scholar awards from the FRQS.

## Disclosures

B.J.A. is a consultant for Novartis and holds/has held research grants from Pfizer, and Ionis Pharmaceuticals. P.M. is a consultant for Casebia Therapeutics. M.L.K. holds/has held research grants from Pfizer; is a member of advisory boards for Sanofi and Amgen; has received speaker’s honoraria/consulting fees from Amgen, Regeneron, and Eli Lilly; and holds/has held research contracts with Sanofi, Ionis, Eli Lilly, and Cardiovax. The remaining authors have no conflicts of interest to disclose.
